# Primary Cilia Mediate TSH-Regulated Thyroglobulin Endocytic Pathways

**DOI:** 10.3389/fendo.2021.700083

**Published:** 2021-09-06

**Authors:** Junguee Lee, Hae Joung Sul, Kun-Ho Kim, Joon Young Chang, Minho Shong

**Affiliations:** ^1^Department of Pathology, Daejeon St. Mary’s Hospital, College of Medicine, The Catholic University of Korea, Seoul, South Korea; ^2^Department of Nuclear Medicine, Chungnam National University Hospital and College of Medicine, Daejeon, South Korea; ^3^Research Center for Endocrine and Metabolic Diseases, Division of Endocrinology, Department of Internal Medicine, Chungnam National University School of Medicine, Daejeon, South Korea

**Keywords:** LRP2/megalin, primary cilium, thyroglobulin endocytosis, ciliogenesis, thyroid follicular cell

## Abstract

Primary cilia are sensory organelles with a variety of receptors and channels on their membranes. Recently, primary cilia were proposed to be crucial sites for exocytosis and endocytosis of vesicles associated with endocytic control of various ciliary signaling pathways. Thyroglobulin (Tg) synthesis and Tg exocytosis/endocytosis are critical for the functions of thyroid follicular cells, where primary cilia are relatively well preserved. LRP2/megalin has been detected on the apical surface of absorptive epithelial cells, including thyrocytes. LRP2/megalin on thyrocytes serves as a Tg receptor and can mediate Tg endocytosis. In this study, we investigated the role of primary cilia in LRP2/megalin expression in thyroid gland stimulated with endogenous TSH using MMI-treated and *Tg-Cre;Ift88^flox/flox^* mice. LRP2/megalin expression in thyroid follicles was higher in MMI-treated mice than in untreated control mice. MMI-treated mice exhibited a significant increase in ciliogenesis in thyroid follicular cells relative to untreated controls. Furthermore, MMI-induced ciliogenesis accompanied increases in LRP2/megalin expression in thyroid follicular cells, in which LRP2/megalin was localized to the primary cilium. By contrast, in *Tg-Cre;Ift88^flox/flox^* mice, thyroid with defective primary cilia expressed markedly lower levels of LRP2/megalin. Serum Tg levels were elevated in MMI-treated mice and reduced in *Tg-Cre;Ift88^flox/flox^* mice. Taken together, these results indicate that defective ciliogenesis in murine thyroid follicular cells is associated with impaired LRP2/megalin expression and reduced serum Tg levels. Our results strongly suggest that primary cilia harbors LRP2/megalin, and are involved in TSH-mediated endocytosis of Tg in murine thyroid follicles.

## Introduction

Thyroglobulin (Tg), the most abundant thyroid-specific protein synthesized by follicular cells (thyrocytes), serves as the molecular template for the synthesis of thyroid hormones T4 and T3 at the thyrocyte–colloid interface. A major regulatory step in thyroid hormone release in mammalian thyroid follicular cells is Tg endocytosis. This process requires micropinocytosis, which includes nonspecific fluid-phase pinocytosis and receptor-mediated endocytosis. Tg internalized by receptors is handled by post-endocytic pathways that sort Tg molecules to undergo lysosomal degradation, transcytosis, or recycling. Effective Tg endocytosis is primarily regulated by TSH and plays an important role in thyroid hormone release. In thyroid follicular cells, clathrin-coated pits, caveolae-dependent endocytosis, and low-density lipoprotein receptor protein 2 (LRP2, also known as megalin) are involved in receptor-mediated endocytic pathways of Tg (1, 2). LRP2/megalin has been detected on the apical surface of thyrocytes; it serves as a Tg receptor and can mediate Tg endocytosis (1, 2). Megalin knockout mice exhibit hypothyroidism, which is associated with reduced levels of serum Tg and free T4 (fT4) levels, and significantly elevated levels of serum TSH (3).

The primary cilia concentrate proteins, hormones, and ions so that they can exert their effects on the primary ciliary membrane. The ciliary pocket, a cytoplasmic invagination of the periciliary membrane, is a crucial site for exocytosis/endocytosis of vesicles for delivery and retrieval of ciliary membrane components; in addition, receptor-mediated endocytosis takes place at the ciliary pocket (4). Previously, we reported that the primary cilia of the mammalian thyroid follicles protrude from the apical surface of follicular cells toward the luminal colloid and present at the cell–colloid interface (5). In murine thyroid follicular cells, the primary cilium plays important roles in maintaining the globular follicular structure of the thyroid (6). Consequently, defective primary cilia in the murine thyroid results in irregular dilation of follicles and colloid Tg depletion (6). Interestingly, the morphological and functional alterations of the thyroid in *Tg-Cre;Ift88^flox/flox^* mice with defective primary cilia resembled those in megalin knockout mice (3).

The primary cilium is the key machinery involved in the transduction of the sonic Hedgehog (Shh) signaling pathway. The components of the Shh pathway, smoothened (Smo), patched 1 (Ptch1), GLI family zinc finger 1 (Gli1), GLI family zinc finger 2 (Gli2), and GLI family zinc finger 3 (Gli3), exhibit dynamic movements along the primary cilium (7, 8). In the central nervous system, the endocytic receptor LRP2/megalin mediates Shh signaling. LRP2/megalin forms a co-receptor complex with Ptch1 that promotes Shh binding and internalization of the Shh/Ptch1 complex and Shh pathway activation in the ciliary pocket of neuroepithelial cells (9). Therefore, we propose that induction of LRP2/megalin-mediated Tg endocytosis is followed by activation of the Shh signaling pathway in primary cilia of thyroid follicular cells.

In this study, we investigated whether the primary cilium of thyroid follicular cells plays a role in Tg endocytosis. We observed the primary cilia or ciliogenesis and LRP2/megalin expression in the murine thyroid gland with endogenous TSH stimulation and high rates of Tg endocytosis. In addition, we observed LRP2/megalin expression in thyroid follicular cells of *Tg-Cre;Ift88^flox/flox^* mice, which have no functional primary cilia due to loss of the *Ift88* gene.

## Materials and Methods

### Mice

Mouse experiments were approved by the Institutional Animal Care and Use Committee of the Catholic Univ. of Korea Daejeon St. Mary’s Hospital (approval ID, CMCDJ-AP-2019-002). Male C57BL/6J mice were purchased from DooYeol Biotech (Seoul, Korea). Mice were housed in temperature-controlled (22 ± 2°C) and light-controlled conditions (12 hours light/12 hours dark cycle, lights on at 7 am), and had free access to food and water. Twelve-week-old C57BL/6J mice were divided into two groups: Group 1 was an untreated control group; Group 2 received 0.05% methimazole (MMI, Sigma-Aldrich, 301507) in distilled drinking water for 4 weeks. MMI is used to establish hypothyroidism in experimental animals. Mouse weights were measured before the start of the experiment and after 4 weeks of MMI exposure.

### Generation of Thyroid-Specific *Ift88*-Knockout Mice

*Ift88^flox/flox^* mice were obtained from Dr. Kim J (Korea Advanced Institute of Science and Technology, Republic of Korea), and *Thyroglobulin-Cre/+* (*Tg-Cre*) transgenic mice were obtained from Dr. Jukka Kero (University of Turku, Finland). The mice were maintained on the C57BL/6 genetic background. *Ift88^flox/flox^* mice were crossed with *Tg-Cre* transgenic mice to generate thyroid follicle-specific *Ift88*-knockout (*Tg-Cre;Ift88^flox/flox^*) mice. Only 35-week-old male mice were used in this study (6 *Tg-Cre;Ift88^flox/flox^* and 6 *Tg-Cre;Ift88^+/+^* mice). The experiments using *Tg-Cre;Ift88* floxed mice received prior approval by the Institutional Animal Care and Use Committee of the Catholic Medical Center (approval ID, CRCC-BE-CMC-17013391).

### Blood Collection and Thyroid Function Test

Retro-orbital blood collected from mice was allowed to clot by leaving the sample undisturbed for 30 minutes at room temperature. The clotted blood was centrifuged at 3000*g* for 10 minutes. Sera were separated and stored at -80°C prior to the hormonal assay. Serum fT4 and serum Tg levels were measured using radioimmunoassay (RIA) by Dr. Kun-Ho Kim (Chungnam National University Hospital, Republic of Korea). Serum TSH was measured using a specific mouse TSH RIA provided by Dr. Cheng SY (Center for Cancer Research, National Cancer Institute, Bethesda, MD, USA).

### Thyroid Extraction

We extracted mouse thyroid using a stereo microscope (Leica EZ4). The right lobe of the dissected thyroid gland was fixed in 10% neutral buffered formalin for 24 hours at room temperature, and then the tissue was embedded in a paraffin block. Tissue slices were subjected to hematoxylin and eosin (H&E) staining and immunohistochemistry. The left lobe of the thyroid gland was stored at -80°C prior to RNA isolation.

### RNA Isolation and RT-qPCR

Total RNA was extracted using TRIzol (Invitrogen). Complementary DNA (cDNA) was synthesized from the total RNA using M-MLV Reverse Transcriptase and oligo-dT primers (Invitrogen). Reverse transcription–quantitative polymerase chain reaction (RT-qPCR) was performed using QuantiTect SYBR Green PCR Master Mix (QIAGEN). Each reaction was performed in triplicate. PCR primers are presented in **Supplementary Table S2**. Amplification conditions were as follows: 10 minutes at 95°C for enzyme activation, followed by 40 cycles of 95°C denaturation for 10 seconds, 60°C annealing for 30 seconds, and 72°C extension for 30 seconds. The cycle threshold (Ct) values for Gapdh RNA and RNA of target genes were measured and calculated using the Life Technologies 7500 software (Life Technologies, Foster City, CA USA). The bar graph data of target genes were normalized to Gapdh mRNA levels.

### Detection of Primary Cilia With Immunofluorescence Staining

Paraffin-embedded 7 μm-thick tissue sections were incubated at 56°C for 5 hours. The sections were then deparaffinized in xylene and rehydrated through a graded series of ethanol baths. Antigens were retrieved in antigen retrieval buffer (0.01 M citric acid–sodium citrate, pH 6.0) by heating the sections in an autoclave at 121°C for 25 minutes. After washing, the sections were air-dried for 30 minutes and rewashed with 1× PBS. The sections were fixed with 4% paraformaldehyde in PBS for 15 minutes and then permeabilized with 0.5% Triton X-100 in PBS for 10 minutes at room temperature. Tissue sections were blocked with 5% bovine serum albumin in PBS for 30 minutes at room temperature. Thereafter, the sections were incubated with primary antibodies for 24 hours at 4°C. On the following day, the tissue-section slides were washed three times with 1× PBS and incubated at 4°C for 12 hours with secondary antibodies. Primary antibodies against acetylated α-tubulin (Cell signaling), ARL13B (ProteinTech Group), polyglutamylation modification (GT335, AdipoGen), γ-tubulin (Sigma-Aldrich), and thyroglobulin (Dako) were used. Goat anti-mouse and goat anti-rabbit secondary antibodies conjugated to Alexa Fluor 488 or 568 (Invitrogen/Life Technologies) were used for indirect immunofluorescence. The stained slides were observed under a FluoView FV1000 microscope equipped with a charge-coupled device camera (Olympus Corp.). The frequency of primary cilia was determined as follows: 100 follicles of similar size were selected; follicular cells with acetylated α-tubulin–positive and γ-tubulin–positive cilia within each thyroid follicle were counted; and the average number of primary cilia per one follicle was calculated.

### Immunohistochemistry Staining

Paraffin-embedded tissue sections (4 μm thick) were incubated at 56°C for 3 hours before immunohistochemistry. The sections were stained using a BenchMark GX automatic system (Ventana Medical Systems, Illkirch, France). All procedures, including antigen retrieval and blocking of endogenous peroxidase activity, were performed automatically by the Benchmark system. Primary antibodies against LRP2 (1:200, Biorbyt), caveolin-1 (1:100, BioVision), Thyroglobulin (1:200, Abcam), Shh (1:100, Bioss Inc.), PTCH1 (1:100, LifeSpan BioSciences), and GLI1 (1:100, Bioss Inc.) were used for immunohistochemistry. Tissue sections were incubated with primary antibody for 32 minutes at 42°C. Immunoperoxidase staining was performed on an LSAB NeuVision system (Ventana) and tissue sections were counterstained with hematoxylin. Tissue slides were analyzed on an OLYMPUS BX51 microscope.

### Transmission Electron Microscopy

The mouse thyroid tissues were fixed in 2.5% glutaraldehyde in 0.1 M cacodylate buffer at 4°C for 4 hours. After washing in 0.1 M cacodylate, samples were post-fixed in 1% OsO_4_ in cacodylate buffer (pH 7.2) containing 0.1% CaCl_2_ for 1 hour at 4°C. Samples were analyzed by electron microscopy (Tecnai G2 Spirit Twin; FEI Company; Korea Basic Science Institute).

### Immunoblot Analysis

Immunoblotting was carried out as previously described (10). The following primary antibodies were used: SHH (Bioss Inc.), PTCH1 (LifeSpan BioSciences), SMO (Abcam), GLI1 (Bioss Inc.), and GAPDH (Abcam).

### Statistical Data Analysis

Serum levels of fT4, TSH, and Tg were assessed by comparing each group to control mice by one-way ANOVA Dunnett’s *post hoc* test. Student’s t-test was used to compare mRNA levels between experimental groups *versus* controls. Data are presented as means ± standard deviation (SD). *P*-value < 0.05 was considered statistically significant.

## Results

### Histopathological and Functional Analysis of Thyroid in Methimazole (MMI)-Treated Mice

To investigate the characteristics of LRP2/megalin expression and Tg endocytosis in the murine thyroid with endogenous TSH stimulation, we observed thyroid follicles and colloid Tg in control and MMI-treated mice.

**Figure 1:**
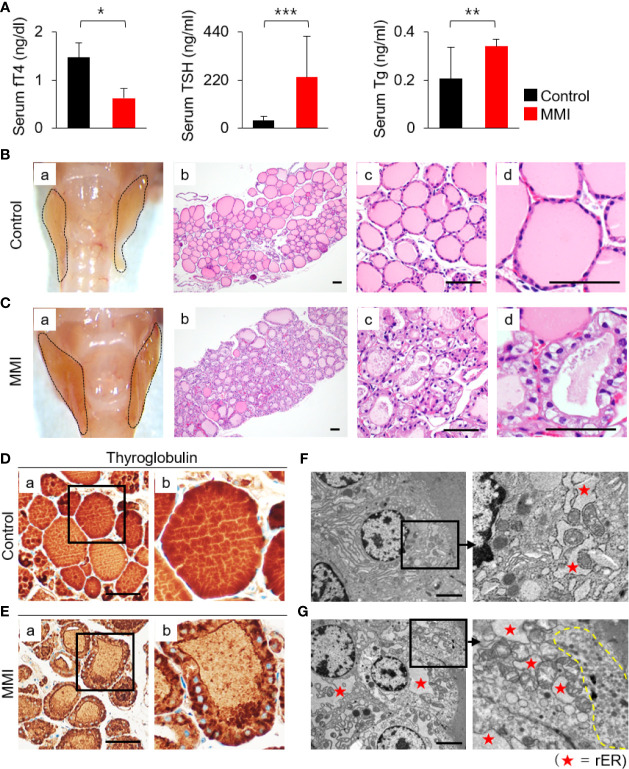
Histopathological characterization and functional activity of thyroid in MMI-treated mice. **(A)** Serum fT4 levels were markedly lower in MMI-treated mice than in control mice (*P* = 0.032). In addition, serum TSH levels in MMI-treated mice were significantly higher (*P* = 0.00004), indicating that primary hypothyroidism in mice was properly induced by MMI treatment. Serum Tg levels were significantly higher in MMI-treated mice (*P* = 0.005). *P < 0.05; **P < 0.01; ***P < 0.001. The size of the thyroid gland was larger in MMI-treated mice than controls **(B-a, C-a)**. **(B)** H&E-stained sections of the control thyroid gland showed round/ovoid follicles containing homogeneous colloids that were regularly distributed. Scale bar, 25 μm. **(C)** In the thyroid of MMI-treated mice, irregularly shaped follicles, consisting of enlarged follicular cells with cytoplasmic vacuoles, had reduced luminal colloids. H&E staining. Scale bar, 25 μm. **(D, E)** Follicular cell activity was analyzed by comparing the concentration of colloid Tg between the thyroid of MMI-treated mice and control groups. Scale bar, 25 μm. **(F, G)** Transmission electron micrographs of thyroid glands containing distended cisternae of the rough endoplasmic reticulum (rER, as indicated by star), as well as numerous endocytic vesicles and electron-dense lysosomes (indicated by dotted line) under the apical plasma membrane, from thyroid follicular cells of MMI-treated mice. Scale bar, 2 μm.

Oral administration of MMI elicited a significant decrease in serum levels of fT4 (control = 1.47 ± 0.31 ng/dL; MMI = 0.63 ± 0.20 ng/dL; *P* = 0.032) and an increase in serum TSH (control = 34.91 ± 20.53 ng/mL; MMI = 232.98 ± 187.91 ng/mL; *P* = 0.00004), resulting in primary hypothyroidism (**Figure 1A** and **Supplementary Table S1**). Serum Tg levels were 0.21 ± 0.13 ng/mL in the control group and 0.34 ± 0.029 ng/mL in the MMI-treated group, respectively. The serum Tg levels were higher in MMI-treated mice than in untreated controls (**Figure 1A**). The thyroid gland and body weight were larger in the MMI-treated group than in the control group (**Figures 1B-a, C-a** and **Supplementary Table S1**).

The thyroid of MMI-treated mice exhibited irregular-shaped follicles, enlarged follicular cells exhibiting cytoplasmic vacuoles with centrally located nuclei, and depletion of luminal colloid (**Figures 1B-b, c, d, C-b, c, d**). Luminal colloid of control thyroid follicles was homogeneously stained by Tg (**Figure 1D-a, b**), whereas in the MMI-treated group, luminal colloid showed reduced Tg staining density (**Figure 1E-a, b**). At times, little to no luminal colloid Tg was observed in the thyroid follicles of MMI-treated mice, yet the cytoplasm of follicular cells was densely stained with Tg (**Figure 1E-b**). These findings indicate that thyroid follicular cells of MMI-treated mice actively take up more colloid Tg due to elevated endogenous TSH stimulation in response to reduced concentrations of serum thyroid hormone, resulting in a smaller follicular lumen, reduced luminal colloid, and elevated cuboidal/columnar follicular cell formation.

Electron microscopic examination of thyroid glands in MMI-treated mice confirmed the increase in cytoplasmic vesicles. More endocytic vesicles and electron-dense lysosomes were found under the apical plasma membrane of thyroid follicular cells in MMI-treated mice than in the control group (**Figures 1F, G**). Further, thyroid follicular cells of MMI-treated mice exhibited hypertrophy relative to the control group (**Figures 1F, G**). In the thyroid follicular cells of MMI-treated mice, the rough endoplasmic reticulum (rER) exhibited distended cisternae, which were visible as cytoplasmic vacuoles under light microscopy (**Figure 1C-d**). Immunohistochemistry revealed cytoplasmic accumulation of Tg in mice treated with MMI (**Figure 1E-b**), but it was not prominent in control mice (**Figure 1D-b**).

Collectively, these morphological changes in MMI-treated mice resulted from increased TSH-stimulated functional activity of thyroid follicular cells.

### Elevated Expression of Lrp2/Megalin in MMI-Treated Mouse Thyroid

**Figure 2:**
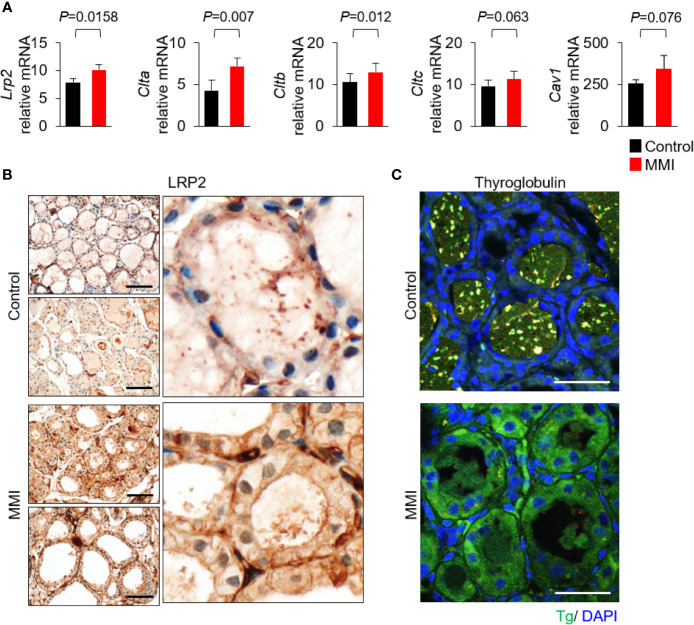
Elevated expression of Lrp2/megalin in MMI-treated mouse thyroid. **(A)** mRNA levels of *Lrp2/megalin* were higher in the thyroid of MMI-treated mice than in the control group (*P* = 0.0158). Two genes associated with Tg endocytosis, *Clta* and *Cltb*, were significantly upregulated in the thyroid of MMI-treated mice relative to the control group (*Clta*, *P* = 0.007; *Cltb*, *P* = 0.012). mRNA levels of *Cav1* (*P* = 0.076) and *Cltc* (*P* = 0.063) were elevated, but the difference was not significant. **(B)** Immunohistochemical staining of LRP2/megalin at the apical plasma membrane of thyroid follicular cells in untreated control mice. In MMI-treated mice, LRP2/megalin was mainly cytoplasmic, but was also detected at the plasma membrane of thyroid follicular cells. Scale bar, 25 μm. **(C)** Immunofluorescence showing localization of Tg. Compared with the control, the level of Tg was markedly higher in the follicular cell cytoplasm of MMI-treated mice. Tg was absent from thyroid follicle lumen of MMI-treated mice but was detectable within the thyroid follicle lumen of the control group. Scale bar, 25 μm.

Based on the morphological and functional characteristics, we investigated whether LRP2/megalin exhibited differential expression under the two experimental conditions. To assess the effects of MMI on receptor-mediated Tg endocytosis of thyroid follicular cells, we first measured the mRNA levels of *Lrp2*/*megalin*, *Clta* (clathrin light chain A), *Cltb* (clathrin light chain B), *Cltc* (clathrin heavy chain), and *Cav1* (caveolin-1). mRNA levels of *Lrp2*/*megalin*, *Clta*, and *Cltb* were higher in the thyroid of MMI-treated mice than the control group (**Figure 2A**). mRNA levels of *Cav1* and *Cltc* were elevated, but not significantly (**Figure 2A**).

Next, we observed the expression pattern of LRP2 in thyroid follicles in mice treated with MMI. LRP2 immunohistochemistry is restricted to the apical plasma membrane of thyroid follicular cells. However, LRP2 expression increased in both the apical plasma membrane and the cytoplasm in follicular cells of the MMI-treated group (**Figure 2B**). Interestingly, Tg immunofluorescence increased in the cytoplasm of follicular cells of MMI-treated mice (**Figure 2C**). These results are consistent with previous observations that Lrp2/megalin mediates Tg uptake under intense TSH stimulation, resulting in transcytosis of Tg from the colloid to the bloodstream (11).

### Increased Ciliogenesis in Thyroid Follicles of MMI-Treated Mice

**Figure 3:**
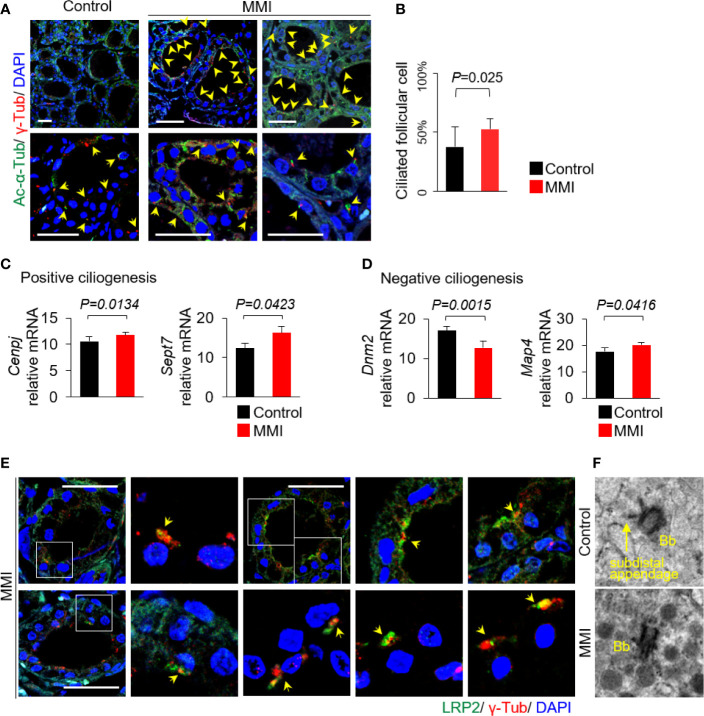
Increased ciliogenesis in thyroid follicles of MMI-treated mice. **(A)** Primary cilia were confirmed by immunofluorescence staining with anti-acetylated α-tubulin (Ac-α-Tub, green) and anti-γ-tubulin (γ-Tub, red). Primary cilia are indicated by arrows. Scale bar, 20 μm. **(B)** Frequency of thyroid follicles containing primary cilia was 37.80 ± 16.75% in the control group and 52.65 ± 9.57% in MMI-treated mice. Ciliogenesis in the thyroid was higher in MMI-treated mice than in control mice (*P* = 0.025). Arrow indicates primary cilia. **(C)** Two genes involved in positive ciliogenesis, *Cenpj* (*P* = 0.0134) and *Sept7* (*P* = 0.0423), were expressed at significantly higher levels in the thyroid of MMI-treated mice than in the control group. **(D)** A gene involved in negative regulation of primary cilia assembly, *Dnm2*, was expressed at significantly lower levels in the thyroid of MMI-treated mice than in the control group (*P* = 0.0015). By contrast, Map4 was upregulated in the thyroid of MMI-treated mice (*P* = 0.0416). **(E)** In thyroid follicular cells of MMI-treated mice, LRP2/megalin (green) localized with γ-tubulin (red, basal body marker), indicating the presence of LRP2/megalin in the basal body of the primary cilium (arrow). Scale bar, 20 μm. **(F)** TEM revealed highly abundant endocytic vesicles and lysosomes primarily located near the basal body (Bb) in thyroid follicles of MMI-treated mice.

We next investigated whether there was an association between LRP2/megalin expression and ciliogenesis of primary cilia *in vivo*. To this end, we performed immunofluorescence analysis to assess changes in ciliogenesis associated with Tg endocytosis. Anti-acetylated α-tubulin and anti-γ-tubulin were used as proteins of the ciliary axoneme and the basal body, respectively (**Figure 3A**). The average frequencies of primary cilia in thyroid of control and MMI-treated mice were 37.80 ± 16.75% and 52.65 ± 9.57%, respectively (**Figure 3B**). Thus, the thyroid follicles in MMI-treated mice exhibited a significant increase in ciliogenesis relative to control thyroids (*P* = 0.025). At the same time, we examined changes in the mRNA expression of genes associated with ciliogenesis. The primary cilium is a dynamic organelle that repeatedly undergoes assembly and disassembly. *Cenpj* (centromere protein J) and *Sept7* (septin 7), which are involved in positive regulation of primary cilia assembly, were expressed at significantly higher levels in the thyroid of MMI-treated mice than in the control group (**Figure 3C**). In addition, a gene encoding a negative regulator of primary cilia assembly, *Dnm2* (dynamin 2), was expressed at lower levels in the thyroid of MMI-treated mice than in the control group. However, *Map4* (microtubule-associated protein 4) was upregulated in the thyroid of MMI-treated mice (**Figure 3D**).

To clarify the relationship between ciliogenesis and LRP2/megalin expression in the thyroid follicles of MMI-treated mice, we evaluated LRP2 localization to primary cilia. Immunofluorescence microscopy revealed that LRP2 was localized with the basal body of primary cilia marked by γ-tubulin (**Figure 3E**). To further confirm the association between primary cilia and Tg endocytosis, we conducted transmission electron microscopy (TEM) in MMI-treated and untreated murine thyroid follicles. Interestingly, abundant endocytic vesicles and lysosomes were primarily located near the basal body in thyroid follicles of MMI-treated mice (**Figure 3F**).

Taken together, endogenous TSH stimulation resulted in increased ciliogenesis and expression of LRP2/megalin in thyroid follicular cells.

### LRP2 Expression of Murine Thyroid Follicles Associated With Ciliogenesis

**Figure 4:**
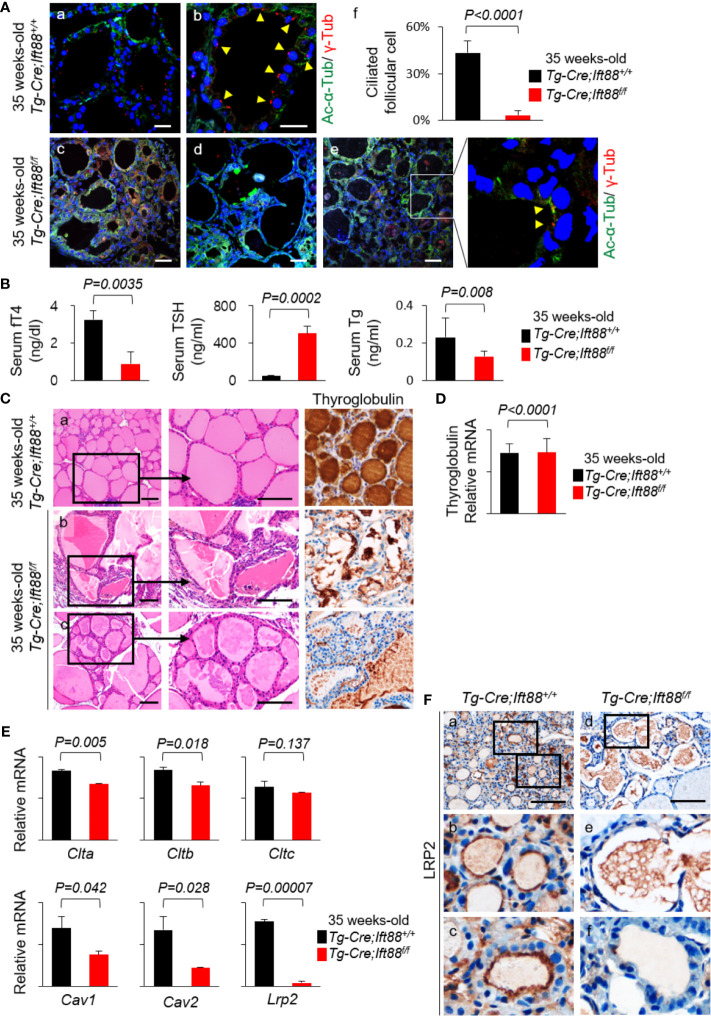
LRP2 expression of murine thyroid follicles regulated by ciliogenesis. **(A)** Immunofluorescence analysis of primary cilia with acetylated α-tubulin (green) to detect the axoneme and γ-tubulin (red) to detect the basal body. Scale bar, 20 μm. Frequency of thyroid follicles containing primary cilia was 3.31 ± 3.00% in the *Tg-Cre;Ift88^flox/flox^* mice and 43.32 ± 7.72% in the *Tg-Cre;Ift88^+/+^* control mice. In the thyroid follicles of *Tg-Cre;Ift88^flox/flox^* mice, primary cilia were rarely detected (*P* < 0.0001). Arrowhead indicates primary cilia. **(B)** Serum fT4 levels were lower in 35-week-old *Tg-Cre;Ift88^flox/flox^* mice (0.89 ± 0.65 ng/dL) than in 35-week-old *Tg-Cre;Ift88^+/+^* control mice (3.25 ± 0.46 ng/dL, *P* = 0.0035). Serum TSH levels were significantly higher in 35-week-old *Tg-Cre;Ift88^flox/flox^* mice (505.31 ± 75.69 ng/mL) than in 35-week-old *Tg-Cre;Ift88^+/+^* mice (53.51 ± 3.25 ng/mL, *P* = 0.0002). Serum Tg levels were significantly lower in 35-week-old *Tg-Cre;Ift88^flox/flox^* mice (0.128 ± 0.026 ng/mL) than in 35-week-old *Tg-Cre;Ift88^+/+^* mice (0.230 ± 0.105 ng/mL, *P* = 0.008). **(C)** Thirty-five-week-old *Tg-Cre;Ift88^flox/flox^* thyroid had irregularly dilated follicles with colloidal Tg-negative lumens. Scale bar, 25 μm. **(D)** There was no difference in thyroid Tg mRNA levels between *Tg-Cre;Ift88^flox/flox^* and *Tg-Cre;Ift88^+/+^* mice (*Tg-Cre;Ift88^flox/flox^* = 14.623 ± 3.327; *Tg-Cre;Ift88^+/+^* = 14.587 ± 2.141; *P* < 0.0001). **(E)** mRNA levels of receptor-mediated Tg endocytosis-related genes *Clta*, *Cltb*, *Cltc*, and *Lrp2* were reduced in 35-week-old *Tg-Cre;Ift88^flox/flox^* thyroid with ciliary loss. mRNA levels of genes associated with caveolae-mediated endocytosis were lower in 35-week-old *Tg-Cre;Ift88^flox/flox^* thyroid than in *Tg-Cre;Ift88^+/+^* control thyroid (*Cav1*, *P*=0.042; *Cav2*, *P*=0.028). **(F)** Immunohistochemical staining of LRP2 was present on the apical plasma membrane of control thyroid follicles, whereas LRP2 was expressed at lower levels in thyroid follicles of 35-week-old *Tg-Cre;Ift88^flox/flox^* mice. Scale bar, 25 μm.

To investigate the *in vivo* functional roles of primary cilia, we recently produced *Tg-Cre;Ift88^flox/flox^* mice to characterize thyroid follicular cell-specific ciliary loss (6). IFT88 is a component of intraflagellar transport particle proteins, which are required for ciliogenesis and ciliary transport (12). We observed the thyroid in *Tg-Cre;Ift88^flox/flox^* mice aged 7, 11, 14, 20, and 35 weeks. The 7, 11, 14, 20, and 35 weeks-old *Tg-Cre;Ift88^flox/flox^* mice exhibited focal histomorphological changes in thyroid follicles. However, 35-week-old *Tg-Cre;Ift88^flox/flox^* mice exhibited profound follicular changes, including irregular dilation. Therefore, we chose 35-week-old mice that were more suitable for this experiment. Frequency of primary cilia per thyroid follicle was 3.31 ± 3.00% in the *Tg-Cre;Ift88^flox/flox^* mice and 43.32 ± 7.72% in the *Tg-Cre;Ift88^+/+^* control mice. Primary cilia were rarely detected in thyroid follicles of *Tg-Cre;Ift88^flox/flox^* mice (*P* < 0.0001) (**Figure 4A**). Thirty-five-week-old *Tg-Cre;Ift88^flox/flox^* mice exhibited hypothyroidism with serum TSH elevation (**Figure 4B**). Serum Tg levels were significantly lower in *Tg-Cre;Ift88^flox/flox^* mice (0.128 ± 0.026 ng/mL) than in Tg-Cre;Ift88+/+ mice (0.230 ± 0.105 ng/mL, *P* = 0.008) (**Figure 4B**).

Histological examinations revealed that ciliary loss mediated by Ift88 deficiency in thyroid follicles of 35-week-old mice caused irregularly shaped follicles with luminal colloid Tg depletion in the entire thyroid (**Figure 4C**). Cytoplasmic Tg was barely detectable in thyroid follicular cells of *Tg-Cre;Ift88^flox/flox^* mice (**Figure 4C**). Here, we determined whether Tg depletion of thyroid follicles in *Tg-Cre;Ift88^flox/flox^* mice was due to an increased endocytosis by high TSH or decreased Tg synthesis. RT-qPCR of Tg mRNA was performed to confirm new Tg synthesis. As a result, there was no difference in thyroid Tg mRNA levels between *Tg-Cre;Ift88^flox/flox^* (14.623 ± 3.327) and *Tg-Cre;Ift88^+/+^* mice (14.587 ± 2.141, *P* < 0.0001) (**Figure 4D**). Therefore, the thyroid colloid Tg depletion in *Tg-Cre;Ift88^flox/flox^* mice was not caused by a decrease in Tg synthesis.

To clarify the relationship between defective primary cilia and Lrp2/megalin expression, we compared the expression of genes related to receptor-mediated Tg endocytosis in 35-week-old *Tg-Cre;Ift88^flox/flox^* thyroid relative to *Tg-Cre;Ift88^+/+^* control thyroids. First, murine thyroids with ciliary loss mediated by Ift88 deficiency exhibited significant downregulation of mRNA levels of *Clta*, *Cltb*, and *Cltc* relative to wild-type controls, although the difference in *Cltc* expression was not statistically significant (**Figure 4D**). mRNA levels of caveolin-1 (*Cav1*) and caveolin-2 (*Cav2*) were lower in the thyroid follicles of *Tg-Cre;Ift88^flox/flox^* than in those of *Tg-Cre;Ift88^+/+^* mice (**Figure 4D**). In particular, mRNA levels of Lrp2/megalin were markedly lower in 35-week-old *Tg-Cre;Ift88^flox/flox^* than *Tg-Cre;Ift88^+/+^* thyroids (**Figure 4E**). Immunohistochemistry revealed that the LRP2 was present on the apical plasma membrane of control thyroid follicles, whereas LRP2 was less expressed in the thyroid follicles of *Tg-Cre;Ift88^flox/flox^* mice (**Figure 4F**).

Therefore, loss of primary cilia in murine thyroid follicles was correlated with reduced expression of Lrp2/megalin, which was also associated with irregularly dilated follicles with colloidal Tg depletion and lower levels of serum Tg.

### Interaction Between LRP2/Megalin, Tg Endocytosis, and SHH Signaling in the Primary Cilium of Thyroid Follicles

**Figure 5:**
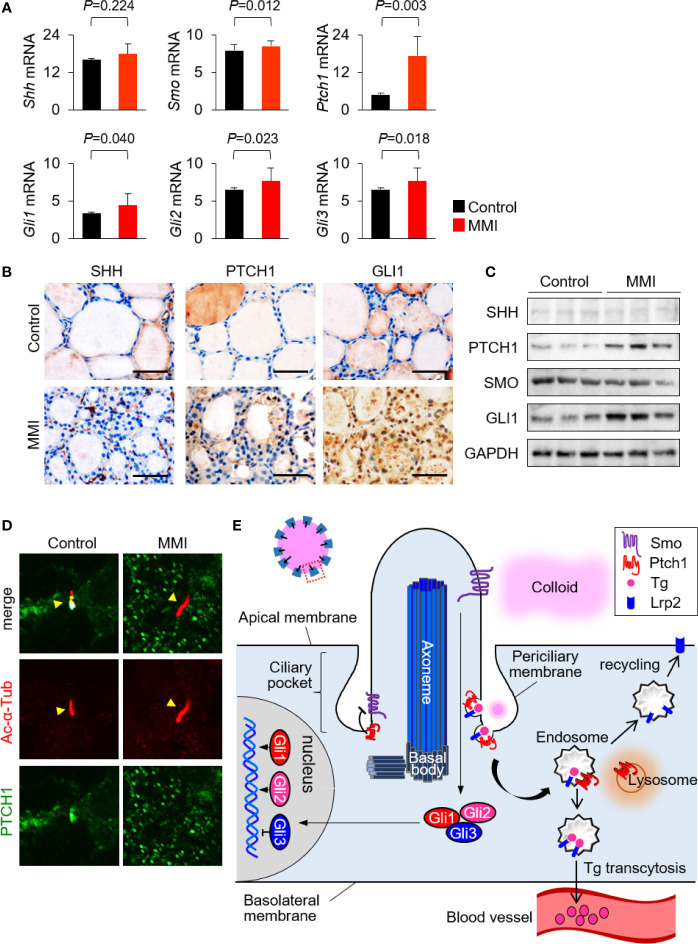
Interaction between LRP2/megalin and SHH in primary cilium of murine thyroid follicles. **(A)** mRNA levels of *Smo*, *Ptch1*, *Gli1*, *Gli2*, and *Gli3* were higher in the thyroid of MMI-treated mice, which exhibited elevated ciliogenesis, than in the control group. **(B)** Immunohistochemistry of PTCH1 and GLI1 was consistently upregulated in the thyroid follicles of MMI-treated mice relative to control. SHH was barely detected in the thyroid of MMI-treated and control mice. Scale bar, 25 μm. **(C)** Immunoblot analysis revealed that PTCH1 and GLI1 levels were higher in MMI-treated mice than in controls. We observed no difference in the expressions of SHH and SMO. Immunoblot data are normalized against GAPDH levels in the same sample. **(D)** PTCH1 was localized in the primary cilium (arrow head) of thyroid follicular cells in the control group. In MMI-treated mice, loss of PTCH1 from the primary cilium (arrow head) was confirmed by immunofluorescence staining. **(E)** Proposed mechanisms for the relationship between receptor-mediated Tg endocytosis and Shh signaling at the primary cilium.

LRP2/megalin-mediated endocytosis controls Shh trafficking and signaling (13–15). The primary cilia, which play critical roles in signal transduction, are central organelles in the Shh signaling pathway (7, 8). The primary cilium can act as a positive or negative regulator of Shh signaling (16, 17). To elucidate the molecular mechanism of LRP2/megalin-mediated Tg endocytosis in primary cilia, we analyzed the expression levels of genes and proteins related to the SHH signaling pathway in the thyroid of MMI-treated and untreated control mice. Although *Shh* mRNA levels exhibited no significant change, the mRNA levels of *Smo*, *Ptch1*, *Gli1*, *Gli2*, and *Gli3* in the thyroid were higher in MMI-treated mice than in the control group (**Figure 5A**). Next, we monitored the expression of SHH signaling pathways in the thyroid of MMI-treated mice by immunohistochemistry and immunoblot assays for SHH, PTCH1, SMO, and GLI1. GLI1 immunohistochemistry was consistently higher in the cytoplasm and nucleus of thyroid follicular cells of MMI-treated mice relative to control, whereas SHH was barely detectable in the thyroid of MMI-treated mice and controls (**Figure 5B**). Immunoblotting revealed an increase of PTCH1 and GLI1 expressions in the thyroid of MMI-treated mice (**Figure 5C**), and it showed an increase in the expression of PTCH1 without a significant change in SHH (**Figure 5C**). These findings indicate that Shh signaling was upregulated in the thyroid of TSH-stimulated mice. Consistent with this, the PTCH1 is detected in primary cilium of controls, whereas it is not observed in primary cilium of MMI-treated mice (**Figure 5D**).

## Discussion

Here, we demonstrated that Lrp2/megalin is localized in the primary cilium of thyroid follicular cells. Endogenous TSH stimulation in MMI-treated mice increased Lrp2/megalin expression and Tg endocytosis. In addition, thyroid-specific cilium-deficient *Tg-Cre;Ift88^flox/flox^* mice exhibited a significant loss of Lrp2/megalin and a reduction in serum Tg despite the high TSH level. Hence, these results may link the functional role of thyroid primary cilium with Lrp2/megalin-mediated Tg endocytosis *in vivo*.

LRP2/megalin plays a role in Tg uptake in thyroid follicular cells. TSH-stimulated Tg uptake is an important determinant of thyroid hormone and Tg release into the bloodstream (18–20). We found that Lrp2/megalin in *Tg-Cre;Ift88^flox/flox^* mice exhibited a significant reduction in serum Tg levels despite having high levels of TSH. These findings indicate that cilium-deficient thyroid follicular cells impaired Tg uptake with TSH stimulation. In this study, we showed that *Tg-Cre;Ift88^flox/flox^* mice developed as irregularly dilated thyroid follicles with depleted colloid Tg. These follicular changes, accompanied by a reduced serum Tg and elevated serum TSH, suggest that absence of LRP2 leads to reduced thyroid hormone and Tg release.

Tg endocytosis can be mediated by a variety of cell membrane proteins, such as asialoglycoprotein receptor (21), N-acetylglucosamine receptor (22), several low-affinity receptors (23, 24) and through components of membrane rafts (25). Therefore, not all the endocytic effects can be directly linked to LRP2/megalin. Nevertheless, reductions of LRP2/megalin and serum Tg in thyroid follicles with ciliary loss make it possible to determine that LRP2/megalin is more suitable for elucidating the association between Tg endocytosis and primary cilia.

We observed a significant increase in Tg endocytosis and serum TSH levels in the thyroid follicular cells of MMI-treated mice. In addition, both the frequency of primary cilia and LRP2/megalin expression were elevated in the thyroid of MMI-treated mice. High TSH stimulation in follicular cells of MMI-treated mice may be responsible for the elevated frequency of primary cilia. TSH is required not only for the differentiated function of thyrocytes, but also for the stimulation of cell cycle progression and proliferation in various species (26). It remains to be determined how TSH stimulation is directly linked to increased ciliogenesis and LRP2/megalin expression of thyroid follicular cells.

In addition, our findings indicate that Shh/Gli1 signaling pathway contributes to Lrp2/megalin-mediated Tg endocytosis in primary cilia of thyroid follicular cells. The co-localization of Lrp2 and Tg promoted activation of Ptch1 and translocation of Gli1 to the nucleus. We speculate that Lrp2/megalin-mediated Tg endocytosis activated by TSH stimulation may promote the internalization of Ptch1 near Lrp2/megalin, alleviating Ptch1-dependent inhibition of Smo activity and activating the Shh signaling pathway (**Figure 5E**).

In conclusion, we have demonstrated that ciliogenesis and LRP2/megalin expression are significantly elevated in the thyroid follicular epithelium of endogenous TSH-stimulated mice. Furthermore, these TSH-stimulated mice showed that LRP2 is localized to the primary cilium. *Tg-Cre;Ift88^flox/flox^* mice, which exhibited thyroid-specific ciliary loss, expressed dramatically lower levels of LRP2/megalin expression. Together, our results strongly suggest that LRP2/megalin-mediated endocytosis of Tg in murine thyroid follicles is regulated by ciliogenesis. This is the first *in vivo* study showing that the primary cilia of thyroid follicular cells are the site of LRP2/megalin-mediated Tg endocytosis.

## Data Availability Statement

The original contributions presented in the study are included in the article/**Supplementary Material**. Further inquiries can be directed to the corresponding authors.

## Ethics Statement

The animal study was reviewed and approved by CMCDJ-AP-2019-002.

## Author Contributions 

JL, JYC, and MS conceived the work, designed and performed the experiments, and wrote the manuscript. HJS provided human thyroid tissue samples. Serum-free thyroxine and Tg levels were measured by K-HK. All authors contributed to the article and approved the submitted version.

## Funding

JL was supported by a grant from the Basic Science Research Program through the National Research Foundation of Korea (NRF) funded by the Ministry of Science, ICT (MISIT) (grant number: NRF-2020R1A2C2010269), and by a grant from the Daejeon St. Mary’s Hospital (grant number: CMCDJ-P-2020-003). This research was supported by a grant from the Korea Health Technology R&D Project through the Korea Health Industry Development Institute (KHIDI), funded by the Ministry of Health and Welfare, Republic of Korea (grant number: HR20C0025).

## Conflict of Interest

The authors declare that the research was conducted in the absence of any commercial or financial relationships that could be construed as a potential conflict of interest.

## Publisher’s Note

All claims expressed in this article are solely those of the authors and do not necessarily represent those of their affiliated organizations, or those of the publisher, the editors and the reviewers. Any product that may be evaluated in this article, or claim that may be made by its manufacturer, is not guaranteed or endorsed by the publisher.

## References

[1] Marino M Zheng G Chiovato L Pinchera A Brown D Andrews D. Role of Megalin (Gp330) in Transcytosis of Thyroglobulin by Thyroid Cells. A Novel Function in the Control of Thyroid Hormone Release. J Biol Chem (2000) 275:7125–37. 10.1074/jbc.275.10.7125 10702280

[2] Marino M Pinchera A McCluskey RT Chiovato L. Megalin in Thyroid Physiology and Pathology. Thyroid (2001) 11:47–56. 10.1089/10507250150500667 11272097

[3] Lisi S Segnani C Mattii L Botta R Marcocci C Dolfi A. Thyroid Dysfunction in Megalin Deficient Mice. Mol Cell Endocrinol (2005) 236:43–7. 10.1016/j.mce.2005.03.009 15878230

[4] Molla-Herman A Ghossoub R Blisnick T Meunier A Serres C Silbermann F. The Ciliary Pocket: An Endocytic Membrane Domain at the Base of Primary and Motile Cilia. J Cell Sci (2010) 123:1785–95. 10.1242/jcs.059519 20427320

[5] Lee J Yi S Kang YE Chang JY Kim JT Sul HJ. Defective Ciliogenesis in Thyroid Hurthle Cell Tumors Is Associated With Increased Autophagy. Oncotarget (2016) 7:79117–30. 10.18632/oncotarget.12997 PMC5346702 27816963

[6] Lee J Yi S Chang JY Kim JT Sul HJ Park KC. Loss of Primary Cilia Results in the Development of Cancer in the Murine Thyroid Gland. Mol Cells (2019) 42:113–22. 10.14348/molcells.2018.0430 PMC6399002 30622229

[7] Corbit KC Aanstad P Singla V Norman AR Stainier DY Reiter JF. Vertebrate Smoothened Functions at the Primary Cilium. Nature (2005) 437:1018–21. 10.1038/nature04117 16136078

[8] Rohatgi R Milenkovic L Scott MP. Patched1 Regulates Hedgehog Signaling at the Primary Cilium. Science (2007) 317:372–6. 10.1126/science.1139740 17641202

[9] Pedersen LB Mogensen JB Christensen ST. Endocytic Control of Cellular Signaling at the Primary Cilium. Trends Biochem Sci (2016) 41:784–97. 10.1016/j.tibs.2016.06.002 27364476

[10] Lee J Yi S Won M Song YS Yi HS Park YJ. Loss-Of-Function of IFT88 Determines Metabolic Phenotypes in Thyroid Cancer. Oncogene (2018) 37:4455–74. 10.1038/s41388-018-0211-6 29743590

[11] Marino M Chiovato L Mitsiades N Latrofa F Andrews D Tseleni-Balafouta S. Circulating Thyroglobulin Transcytosed by Thyroid Cells in Complexed With Secretory Components of Its Endocytic Receptor Megalin. J Clin Endocrinol Metab (2000) 85:3458–67. 10.1210/jc.85.9.3458 10999849

[12] Katoh Y Terada M Nishijima Y Takei R Nozaki S Hamada H. Overall Architecture of the Intraflagellar Transport (IFT)-B Complex Containing Cluap1/IFT38 as an Essential Component of the IFT-B Peripheral Subcomplex. J Biol Chem (2016) 291:10962–75. 10.1074/jbc.M116.713883 PMC4900248 26980730

[13] McCarthy RA Barth JL Chintalapudi MR Knaak C Argraves WS. Megalin Functions as an Endocytic Sonic Hedgehog Receptor. J Biol Chem (2002) 277:25660–7. 10.1074/jbc.M201933200 11964399

[14] Morales CR Zeng J El Alfy M Barth JL Chintalapudi MR McCarthy RA. Epithelial Trafficking of Sonic Hedgehog by Megalin. J Histochem Cytochem (2006) 54:1115–27. 10.1369/jhc.5A6899.2006 PMC3957805 16801528

[15] Ortega MC Cases O Merchan P Kozyraki R Clemente D de Castro F. Megalin Mediates the Influence of Sonic Hedgehog on Oligodendrocyte Precursor Cell Migration and Proliferation During Development. Glia (2012) 60:851–66. 10.1002/glia.22316 22354480

[16] Wheway G Nazlamova L Hancock JT. Signaling Through the Primary Cilium. Front Cell Dev Biol (2018) 6:8. 10.3389/fcell.2018.00008 29473038 PMC5809511

[17] Goetz SC Ocbina PJ Anderson KV. The Primary Cilium as a Hedgehog Signal Transduction Machine. Methods Cell Biol (2009) 94:199–222. 10.1016/S0091-679X(08)94010-3 20362092 PMC2867239

[18] Romagnoli P Herzog V. Transcytosis in Thyroid Follicle Cells: Regulation and Implications for Thyroglobulin Transport. Exp Cell Res (1991) 194:202–9. 10.1016/0014-4827(91)90355-X 2026176

[19] Herzog V. Transcytosis in Thyroid Follicle Cells. J Cell Biol (1983) 97:607–17. 10.1083/jcb.97.3.607 PMC2112552 6885913

[20] Herzog V. Pathways of Endocytosis in Thyroid Follicle Cells. Int Rev Cytol (1984) 91:107–39. 10.1016/S0074-7696(08)61315-7 6389418

[21] Stockert RJ. The Asialoglycoprotein Receptor: Relationships Between Structure, Function, and Expression. Physiol Rev (1995) 75:591–609. 10.1152/physrev.1995.75.3.591 7624395

[22] Consiglio E Shifrin S Yavin Z Ambesi-Impiombato FS Rall JE Salvatore G. Thyroglobulin Interactions With Thyroid Membranes. Relationship Between Receptor Recognition of N-Acetylglucosamine Residues and the Iodine Content of Thyroglobulin Preparations. J Biol Chem (1981) 256:10592–9. 10.1016/S0021-9258(19)68664-3 6270119

[23] Giraud A Siffroi S Lanet J Franc JL. Binding and Internalization of Thyroglobulin: Selectivity, pH Dependence, and Lack of Tissue Specificity. Endocrinology (1997) 138:2325–32. 10.1210/endo.138.6.5195 9165018

[24] Lemansky P Herzog V. Endocytosis of Thyroglobulin Is Not Mediated by Mannose-6-Phosphate Receptors in Thyrocytes. Evidence for Low-Affinity-Binding Sites Operating in the Uptake of Thyroglobulin. Eur J Biochem (1992) 209:111–9. 10.1111/j.1432-1033.1992.tb17267.x 1396689

[25] Luo Y Akama T Okayama A Yoshihara A Sue M Oda K. A Novel Role for Flotillin-Containing Lipid Rafts in Negative-Feedback Regulation of Thyroid-Specific Gene Expression by Thyroglobulin. Thyroid (2016) 26:1630–9. 10.1089/thy.2016.0187 27676653

[26] Lee YJ Park DJ Shin CS Park KS Kim SY Lee HK. Microarray Analysis of Thyroid Stimulating Hormone, Insulin-Like Growth Factor-1, and Insulin-Induced Gene Expression in FRTL-5 Thyroid Cells. J Korean Med Sci (2007) 22:883–90. 10.3346/jkms.2007.22.5.883 PMC2693858 17982240

