# Supplementary material for: Primary Cilia Mediate TSH-Regulated Thyroglobulin Endocytic Pathways
Source: Front Endocrinol (Lausanne). 2021 Sep 6;12:700083. doi: 10.3389/fendo.2021.700083 (PMC8451241; doi:10.3389/fendo.2021.700083)
Supplement: Supplementary file 1 [file Table_1.pdf]

Supplementary Information

Primary cilia mediate TSH-regulated thyroglobulin endocytic pathways

Junguee Lee[1]*, Hae Joung Sul[1], Kun-Ho Kim[2], Joon Young Chang[3], Minho Shong[3]*

[1]Department of Pathology, Daejeon St. Mary's Hospital, College of Medicine, The Catholic University of Korea, Seoul, Republic of Korea
[2]Department of Nuclear Medicine, Chungnam National University Hospital and College of Medicine, Daejeon, Republic of Korea
[3]Research Center for Endocrine and Metabolic Diseases, Division of Endocrinology, Department of Internal Medicine, Chungnam National University School of Medicine, Daejeon, Republic of Korea

***Corresponding author:**

Junguee Lee, MD, PhD
junguee@catholic.ac.kr

Minho Shong, MD, PhD
minhos@cnu.ac.kr

SUPPLEMENTARY TABLES

Supplementary Table S1. Serum levels of TSH and FT4 in MMI-treated mice and controls.

		Body weight (g)		Serum TSH (ng/ml)	Serum FT4 (ng/dl)
		12-week-aged	16-week-aged		
C1	No treatment	23.16	27.08	17.75	1.92
C2		23.91	25.84	49.76	1.39
C3		24.36	27.48	55.39	1.33
C4		23.26	24.14	16.74	1.24
mean ± SD		23.67 ± 0.57	26.14 ± 1.50	34.91 ± 20.53	1.47 ± 0.31
MMI-1	MMI 0.05%	23.88	23.51	73.01	0.87
MMI-2		23.92	22.43	78.51	0.85
MMI-3		23.53	22.84	228.82	0.52
MMI-4		23.64	21.57	230.02	0.52
MMI-5		23.48	21.92	90.45	0.75
MMI-6		23.50	23.13	80.12	0.82
MMI-7		23.00	21.79	481.32	0.46
MMI-8		23.44	21.71	244.52	0.51
MMI-9		23.46	22.27	590.08	0.34
mean ± SD		23.54 ± 0.27	22.35 ± 0.68	232.98 ± 187.91	0.63 ± 0.20

Reference values of FT4 (0.70–1.90 ng/dl).

Supplementary Table S2. Lists of primer pairs used in qRT-PCR

Gene	Forward	Reverse
Lrp2	5′-CCTTGCCAAACCCTCTGAAAAT-3′	5′-CACAAGGTTTGCGGTGTCTTTA-3′
Clta	5′-ATGCTGTTGACGGAGTGATGA-3′	5′-CCACTTACGGATACTTTCAGGCT-3′
Cltb	5′-GAAAGCGAGATTGCTGGCATC-3′	5′-CGTTAGCCTCCTGAAACACATC-3′
Cltc	5′-AGATTCTGCCCATTCGCTTTC-3′	5′-TCAGTGCAATCACTTTGCTGG-3′
Cav1	5′-CACACCAAGGAGATTGACCTGG-3′	5′-CCTTCCAGATGCCGTCGAAACT-3′
Cav2	5′-ATGACGCCTACAGCCACCACAG-3′	5′-GCAAACAGGATACCCGCAATG-3′
Sept7	5′-GAAGGTGGTGTTCAGTTGCTGC-3′	5′-GCATCTGACGTCTGTTCACTCG-3′
Cenpj	5′-GCAAGAGAAGCTGCTCACCATG-3′	5′-GTAGCTGTGTGGTGGCAGAGTT-3′
Dnm2	5′-ATGCTGCCACTGGACAACCTCA-3′	5′-GCCAGTTCAATCTGTCGAAGGTC-3′
Map4	5′-ACAGTACCTCCTTGCACGGCTT-3′	5′-CCAGGCGTTTCCTTCTGCTCTA-3′
Shh	5′-CCACTGTTCTGTGAAAGCAGAG-3′	5′-CAGCGTCTCGATCACGTAGAAG-3′
Smo	5′-CTGACTGGCGGAACTCCAATCG-3′	5′-CAGACTACTCCAGCCATCAAGG-3′
Ptch1	5′-CCCTAACAAAAATTCAACCAAACCT-3′	5′-GCATATACTTCCTGGATAAACCTTGAC-3′
Gli1	5′-GCCACACAAGTGCACGTTTG-3′	5′-AAGGTGCGTCTTGAGGTTTTCA-3′
Gli2	5′-TTTGCCGATTGACATGAGACA-3′	5′-GGTGGGAGGCCCGTGTAC-3′
Gli3	5′-CGCAGGTCTGTGGATTTGG-3′	5′-CTCATTACTTCAGCTTGTTTCTCAAAG-3′
Gapdh	5′-CATCACTGCCACCCAGAAGACTG-3′	5′-ATGCCAGTGAGCTTCCCGTTCAG-3′
